# Designing a valid randomized pragmatic primary care implementation trial: the my own health report (MOHR) project

**DOI:** 10.1186/1748-5908-8-73

**Published:** 2013-06-25

**Authors:** Alex H Krist, Beth A Glenn, Russell E Glasgow, Bijal A Balasubramanian, David A Chambers, Maria E Fernandez, Suzanne Heurtin-Roberts, Rodger Kessler, Marcia G Ory, Siobhan M Phillips, Debra P Ritzwoller, Dylan H Roby, Hector P Rodriguez, Roy T Sabo, Sherri N Sheinfeld Gorin, Kurt C Stange

**Affiliations:** 1 Department of Family Medicine and Population Health, Virginia Commonwealth University, PO Box 980251, Richmond, VA 23298-0251, USA; 2 Department of Health Policy & Management, UCLA Fielding School of Public Health, Los Angeles, CA 90095-1772, USA; 3 Implementation Science Team, Division of Cancer Control and Population Sciences, National Cancer Institute, 6130 Executive Blvd, Room 6144, Rockville, MD 20852, USA; 4 Division of Epidemiology, Human Genetics, and Environmental Science, University of Texas Health Science Center at Houston, School of Public Health, 5323 Harry Hines Blvd, Dallas, TX 75390, USA; 5 Division of Services and Intervention Research, National Institute of Mental Health, 6001 Executive Blvd., Room 7144, Bethesda, MD 20892-9631, USA; 6 University of Texas Health Science Center at Houston, School of Public Health, 7000 Fannin Suite 2558, Houston, TX 77030, USA; 7 Department of Family Medicine, Vermont College of Medicine, S458 Courtyard at Given, 89 Beaumont Ave., Burlington, VT 05405, USA; 8 Department of Health Promotion and Community Health Sciences, Texas A&M Health Sciences Center School of Rural Public Health, College Station, TX 77843-1266, USA; 9 Institute for Health Research, Kaiser Permanente Colorado, 10065 E. Harvard Ave., Denver, CO 80231, USA; 10 Department of Biostatistics, Virginia Commonwealth University, PO Box 980032, Richmond, VA 23298-0032, USA; 11 SAIC-Frederick, NIH, NCI, DCCPS, 6130 Executive Plaza, Bethesda, MD, USA; 12 New York Physicians against Cancer (NYPAC), Herbert Irving Comprehensive Cancer Center, New York, NY, USA; 13 Family Medicine & Community Health, Epidemiology & Biostatistics, Sociology and Oncology, Case Western Reserve University, 11000 Cedar Ave, Suite 402, Cleveland, OH 44106, USA

**Keywords:** Patient-reported measures, Health behaviors, Mental health, Pragmatic trial, Electronic health record, Primary care

## Abstract

**Background:**

There is a pressing need for greater attention to patient-centered health behavior and psychosocial issues in primary care, and for practical tools, study designs and results of clinical and policy relevance. Our goal is to design a scientifically rigorous and valid pragmatic trial to test whether primary care practices can systematically implement the collection of patient-reported information and provide patients needed advice, goal setting, and counseling in response.

**Methods:**

This manuscript reports on the iterative design of the My Own Health Report (MOHR) study, a cluster randomized delayed intervention trial. Nine pairs of diverse primary care practices will be randomized to early or delayed intervention four months later. The intervention consists of fielding the MOHR assessment – addresses 10 domains of health behaviors and psychosocial issues – and subsequent provision of needed counseling and support for patients presenting for wellness or chronic care. As a pragmatic participatory trial, stakeholder groups including practice partners and patients have been engaged throughout the study design to account for local resources and characteristics. Participatory tasks include identifying MOHR assessment content, refining the study design, providing input on outcomes measures, and designing the implementation workflow. Study outcomes include the intervention reach (percent of patients offered and completing the MOHR assessment), effectiveness (patients reporting being asked about topics, setting change goals, and receiving assistance in early versus delayed intervention practices), contextual factors influencing outcomes, and intervention costs.

**Discussion:**

The MOHR study shows how a participatory design can be used to promote the consistent collection and use of patient-reported health behavior and psychosocial assessments in a broad range of primary care settings. While pragmatic in nature, the study design will allow valid comparisons to answer the posed research question, and findings will be broadly generalizable to a range of primary care settings. Per the pragmatic explanatory continuum indicator summary (PRECIS) framework, the study design is substantially more pragmatic than other published trials. The methods and findings should be of interest to researchers, practitioners, and policy makers attempting to make healthcare more patient-centered and relevant.

**Trial registration:**

Clinicaltrials.gov: NCT01825746

## Background

There is significant interest in transforming health information technology to be more patient-centered [1-3]. To further integrate these movements toward patient-centeredness and information technology-supported healthcare, one key element that has been missing is a set of brief, practical patient-reported items that are relevant to and actionable by patients and their healthcare team [4,5]. In 2011, 93 national primary care, public health, health behavior, and psychosocial experts as well as patients engaged in a rigorous three-phase process to identify evidence-based, patient-reported measures that if routinely collected could be used to improve health and monitor health status [6]. The process included assembling all possible candidate measures, using a grid-enabled measures wiki platform to identify key measures for consideration [7], and a town hall meeting to finalize the measures. From this process, 17 brief, feasible screening questions evaluating 10 domains of health behaviors and psychosocial problems were identified [8].

Before these patient-reported measures are fully integrated into health risk assessments or routinely integrated into electronic health records (EHRs) and patient portals, research is needed to evaluate relevance, feasibility, and impact on care [9-11]. Randomized pragmatic implementation studies are one way to efficiently and rapidly make this evaluation [12]. Key to pragmatic studies is stakeholder involvement, of both clinicians and patients, and outcomes important to potential adoption settings. Table 1 summarizes the key characteristics of pragmatic trials compared to traditional efficacy trials in terms of measures, costs, focus and other dimensions. As shown, there are major differences in the formulation of study questions, methods issues receiving the greatest priority, outcomes and analysis, and level of stakeholder involvement. Despite the differences, if properly designed, both study designs can yield scientifically valid answers to posed research questions. The Consolidated Standards of Reporting Trials (CONSORT) group has also developed a reporting standard and a helpful summary figure, called the Pragmatic Explanatory Continuum Indicator Summary (PRECIS), to report the extent to which an intervention study is pragmatic versus explanatory on 10 dimensions [13,14].

**Table 1:** Distinguishing differences between pragmatic and traditional clinical efficacy trials

	**Pragmatic study**	**Traditional clinical efficacy**
Stakeholder involvement	Engaged in all study phases including study design, conducting the study, collecting data, interpreting results, disseminating findings	Limited engagement, often in response to investigator ideas or study subjects
Research Design	Includes internal and external validity, design fidelity and local adaptation, real life settings and populations, contextual assessments	Focus on limiting threats to internal validity, typically uses randomized controlled trial, participants and settings typically homogenous
Outcomes	Reach, effectiveness, adoption, implementation, comparative effectiveness, sustainability	Efficacy, mechanism identification, component analysis
Measures	Brief, valid, actionable with rapid clinical utility, feasible in real world and low-resource settings	Validated measures that minimize bias, focus on internal consistency and theory rather than clinical relevance
Costs	Assessments include intervention costs and replication costs in relation to outcomes	Often not collected or reported
Data Source	May include existing data (electronic health records, administrative data) and brief patient reports.	Data generation and collection part of clinical trial
Analyses	Process and outcome analyses relevant to stakeholders and from different perspectives	Specified a priori and typically restricted to investigator hypotheses
Availability of findings	Rapid learning and implementation	Delay between trial completion and analytic availability

The purposes of this manuscript are to describe the background, engagement of stakeholders, characteristics of participating primary care practices, study design in relation to the pragmatic explanatory continuum, key outcomes, and automated tool and feedback system developed for the My Health Outcomes Report (MOHR) study.

## Methods

### Overall design

**Figure 1:**
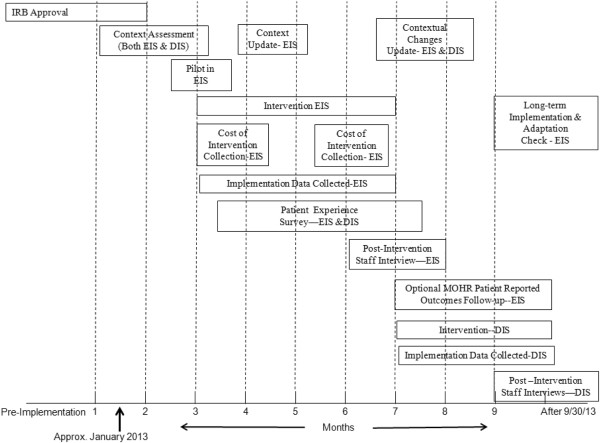
**MOHR study overview and timeline.** The study started in January 2013. The approximate implementation date for the early implementation practices is March 2013 and July 2013 for the delayed implementation practices. (IRB = institutional review board, EIS = early implementation sites, DIS = delayed implementation sites).

The MOHR study is a paired, cluster (practice level) randomized, non-blinded, practical, implementation study that uses a delayed intervention design with 9 pairs of primary care practices (18 total). The design combines elements of pragmatic trials, implementation science, systems science, and mixed methods approaches, and engages stakeholders as partners throughout the study design, implementation, evaluation and subsequent dissemination. One practice in each pair will be randomized to early implementation, and the second to delayed implementation four months later. Implementation will consist of each practice defining a patient population and workflow and then fielding the systematic collection of the patient reported measures in daily care. The primary outcomes will be implementation measures and costs that are relevant to potential adopting and funding agencies [15-19]. Patient goal-setting and clinical follow-up in the 10 health behavior and psychosocial domains will be compared between early and delayed intervention sites prior to implementation of the MOHR assessment in delayed intervention sites, thus delayed intervention practices effectively serve as control practices. Feasibility will be assessed by monitoring implementation in both early and delayed implementation sites (see Figure 1 for project overview and timeline). The project is funded through a unique partnership of grant supplementation from the National Cancer Institute (NCI), the Agency for Healthcare Research and Quality (AHRQ), and the Office of Behavioral Social Sciences Research (OBSSR).

### Research teams and study settings

**Figure 2:**
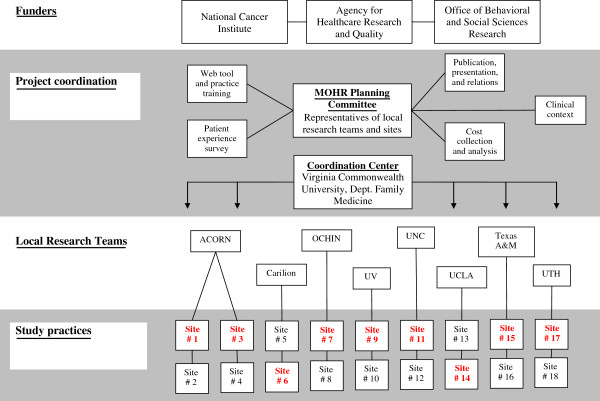
**MOHR study organization and coordination.** The four general partners include funders, project coordination, local research teams, and study practices. The funders provide project coordinators general input into the study implementation and design. Overall project coordination includes the five working groups, MOHR planning committee, and the coordination center. The local research teams directly coordinate with the nine practice pairs to carry out and support the MOHR implementation and evaluation. (ACORN = Virginia Ambulatory Care Outcomes Research Network, UV = University of Vermont, UNC = University of North Carolina, UCLA = University of California, Los Angeles, and UTH = University of Texas Houston).

To carry out this pragmatic trial, partnerships were created between local research teams at eight nationally distributed academic institutions that manage a practice-based research network (PBRN) or participate in the Cancer Prevention and Control Research Network (CPCRN) [20,21]. Given the network affiliations, each research team has expertise in partnering with their practice and patient stakeholders. Accordingly, research teams are charged with helping their partner practices implement the patient-reported assessment and carry out the MOHR study protocol, while balancing the need for adaptation to ensure implementation is relevant to local culture and practice flow (see ‘Balancing fidelity and adaption,’ below) [22]. To coordinate activities across the project, a MOHR planning committee composed of representatives from all local research teams and practices will have joint bimonthly meetings for planning (pre-implementation) and to share successes and challenges (post-implementation). The organization and coordination of activities is depicted in Figure 2.

Research teams identified one or two pairs of primary care practices within their network that were similar with respect to practice type (*e.g.*, FQHC or PBRN, family practice or internal medicine), practice ownership, geographic region, EHR infrastructure, and patient population served. Practice pairs were purposefully selected to represent the diversity of primary care settings and populations to ensure greater generalizability of results. Practices were randomly allocated to the early or delayed condition by coin toss. To conceal allocation, the researchers randomizing practices *(i.e.*, project coordination members) were blinded to practice name; practices were labeled by number in paired blocks (by local research teams), and identity was revealed to all participants one week later during a joint call.

Table 2 presents the characteristics of the 18 participating practices. The PBRN practices tend to have higher proportions of insured patients and fewer ethnic minorities compared to the FQHCs. Most practices are small to medium in size, with one to six providers and seeing 1,500 to 10,000 unique adult patients annually. All practices, except two, have EHRs. While nearly half of practices have initiated or received patient-centered medical home designations, most report limited experience with behavioral health and varying levels of experience with quality improvement.

**Table 2:** Participating practice characteristics

**Site**	**State**	**Setting**	**Patients seen per year**	**Provider FTEs**	**Rooming staff FTE**	**Patient ethnicity and race**		**Insurance type**		
						**Latino**	**Black**	**Medicare**	**Medicaid**	**None**
1	VA	S	5,000	2	4	15%	10%	5%	0%	30%
2	VA	S	1,500	1	2	20%	10%	1%	0%	9%
3	VA	R	2,500	1.6	7	1%	49%	12%	1%	49%
4	VA	S	5,200	4	11	2%	18%	15%	2%	18%
5	VA	U	3,700	5.9	17.3	2%	39%	24%	2%	39%
6	VA	U	3,400	5.3	16.9	1%	17%	26%	1%	17%
7	CA	R	3,500	5.5	15	3%	1%	13%	3%	1%
8	CA	R	5,400	7	25.5	13%	2%	12%	13%	2%
9	VT	R	9,500	5	13.5	1%	5%	13%	1%	5%
10	VT	R	10,000	5	14	1%	2%	15%	1%	2%
11	NC	R	1,100	4.5	12	2%	60%	49%	2%	60%
12	NC	R	7,500	3.5	10	40%	60%	10%	10%	70%
13	CA	U	2,040	1	7	75%	25%	5%	45%	50%
14	CA	U	2,180	2	6	75%	25%	5%	45%	50%
15	TX	R	4,800	2	6	48%	23%	2%	48%	23%
16	TX	R	3,800	2	6	23%	32%	2%	23%	32%
17	TX	U	2,800	3	19	82%	6%	1%	82%	6%
18	TX	U	2,800	3.6	12	80%	5%	1%	80%	5%

Notes:

Practices 1-10 belong to a practice based research network and practices 11-18 belong to the Cancer Prevention and Control Research Network.

All practices except Site 2 and 3 have an electronic health record.

All practices have experience with prior research or quality improvement projects.

*FTE* Full Time Equivalent.

*S* Suburban, *R* Rural, *U* Urban.

### Work groups

To facilitate rapid, collaborative decision making on important issues relevant to the MOHR study design and implementation, work groups were formed consisting of research team members, clinicians, funding agency representatives, and content experts. Each work group is responsible for effectively solving issues related to their specific MOHR study area and bringing proposals to the larger collaborative for approval. Work groups include: Web Tool and Practice Training Work Group – to design and test an electronic version of the comprehensive behavioral and psychosocial risk assessment, http://www.MyOwnHealthReport.org, for practices to use and to create training sessions to prepare practices to address topics identified by the MOHR assessment; Patient Experience Survey Work Group – to design the Patient Experience Survey (see ‘Outcomes assessment’ , below) for assessing main patient-level outcomes; Clinical Context Work Group – to identify and evaluate how key practice, patient, and environmental contextual issues impact implementation; Cost Collection and Analyses Work Group – to measure the practice costs of implementing the MOHR assessment; and Publication, Presentation, and Relations Work Group – to promote opportunities for participants to present and publish findings, coordinate with relevant stakeholder entities, and create a repository of materials, tools, and disseminated findings.

### Intervention

The intervention being studied is the systematic implementation and fielding of the MOHR health behavior and psychosocial assessment, including the delivery of brief customized patient and provider feedback reports. Each local research team worked with their practice pair champions and key stakeholders to determine the most effective workflow for administering the MOHR assessment, including: (a) which patients would be invited to complete the assessment, (b) when the assessment would be completed (*e.g.*, two weeks before a visit, immediately before a visit), (c) where the assessment would be completed (home or practice), (d) whether the assessment would be electronic or paper based, and (e) how clinicians would receive MOHR feedback summaries within the context of office visits. This is consistent with the original NIH consensus panel’s goal of identifying and standardizing patient reported elements to be incorporated into EHRs while minimizing staff burden and enhancing consistency of implementation. As a result, the practice pairs were encouraged to field the electronic version of the MOHR assessment if possible [8].

Because it was not feasible to fully integrate the MOHR assessment into each of the diverse practice’s EHR and patient portal, a stand alone, publically available website (http://www.MyOwnHealthReport.org) was created. The MOHR website is partially integrated with the study practices’ existing EHRs and administers the 17 health behavior and psychosocial screening questions (including basic demographics) as defined by the NIH consensus panel [6,8]. In response to positive depression, anxiety, alcohol, and drug screening questions, the website seamlessly prompts the patient to complete more in-depth assessments, including the Patient Health Questionnaire (PHQ9) [23], the Generalized Anxiety Disorder (GAD) questionnaire [24,25], the Alcohol Use Disorders Identification Test (AUDIT-C) [26], and the Drug Abuse Screening Test (DAST-10) [27], respectively. The MOHR website scores the patient’s responses and characterizes them as being of ‘no concern’ , ‘some concern’, or ‘high concern’ based on norms and national guidelines. For measures with ‘some’ or ‘high concern’ , patients are asked to select which topics they are ready to change and/or discuss with their doctor and select the one topic they feel is most important to address [28-31].

**Figure 3:**
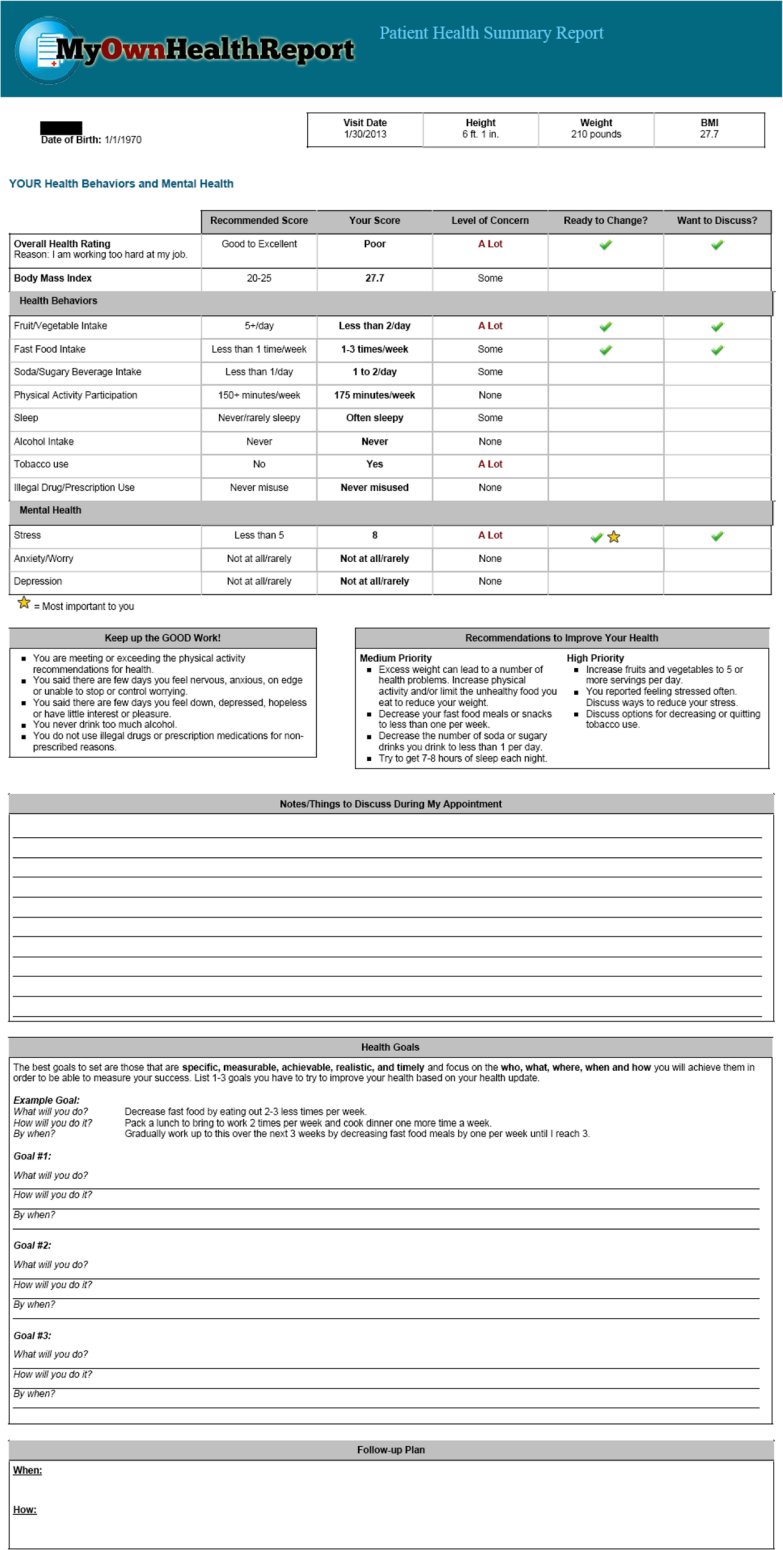
**Patient summary and feedback from **http://www.MyOwnHealthReport.org**.** The first page demonstrates the patient’s health behavior and psychosocial scores, level of concern, and whether the patient reported readiness to change and interest in talking with their doctor. The second page includes patient workspace for notes, creation of SMART health goals, and a follow-up plan.

After completing this process, patients are taken to a feedback page to review, download and print (see Figure 3). Health domains in which they are doing well are highlighted and reinforced, initial recommendations for changes and improvements are given for domains where improvements are needed, and space is provided to list any questions, decide on and create up to three SMART goals (specific, measurable, achievable, realistic and timely), and outline follow-up steps to help achieve their goals [32,33]. When patients have finished reviewing their feedback page and leave the website, a clinician summary is also sent to the patient’s primary care team.

### Practice implementation preparation

Practices are being prepared to implement the MOHR assessment through a series of calls and meetings with the site champions, an educational webinar with an interactive ‘call in’ and case scenarios, support materials, and on-site consultation for follow-up. The webinars have been designed using the principles of adult learning, including multiple modalities, reinforcement, modeling and observation. The follow-up consultations have been modeled on academic detailing that entails a brief face-to-face intervention with the clinician, repeated at periodic intervals [34-36]. Academic detailing has been found effective in improving other aspects of clinical care such as cancer screening and chronic disease management [37-43].

The webinars present the evidence supporting the MOHR assessment, strategies for successful practice implementation of risk assessments, and approaches to counseling and supporting patients as they address identified problem behaviors and psychosocial issues. The webinars, as academic detailing, distill the evidence into brief, practical messages that are relevant to and actionable for primary care clinicians. The webinar and local research team consultations seek to change staff attitudes and beliefs toward implementing the MOHR assessment though persuasive communications, including case examples, tailored feedback and reinforcement [44]. Feedback and reinforcement will include weekly practice reports on the number of patients completing the MOHR assessment and bimonthly meetings between practice champions and local research teams to share cross project implementation experiences and review the local implementation process.

### Data sources

Outcomes will be collected from three sources: practice appointment records, the Patient Experience Survey, and patient responses to the MOHR assessment. Appointment records will be used for calculating Reach (see ‘Primary outcomes’, below). Reach measures are collected when practices enter the intervention phase, months two to six for early intervention practices and months seven to nine for delayed intervention practices. Appointment records are queried by the practices (electronically or manually) for patients who meet inclusion criteria, are documented, de-identified, and transferred to a central data coordination center every two weeks. The Patient Experiences Survey will be used to compare the early versus delayed intervention practices on patient reports of patient-provider discussions, recommendations for referrals, and collaborative goal-setting across the 10 health domains. The patient responses to the MOHR assessment will be used for calculating patient health behavior and psychosocial changes over time. This will be assessed by repeated administration of the MOHR assessment for the cohort of patients who complete the assessment on the website and provide email addresses (see ‘Outcomes assessment’ , below).

The content of the Patient Experience Survey and fielding protocol were developed by the working group, building on earlier pilot phase experiences and practice input, with the intention of balancing scientific rigor and patient needs, practice capabilities, and resource availability across the diverse settings. The survey questions were standardized. The 5As framework (Assess, Advise, Agree, Assist and Arrange) served as a guide for developing questions for each health domain [45]. Questions from the Consumer Assessment of Healthcare Providers and Systems (CAHPS) assessing patient trust in their healthcare team and perceived provider communication style were also included [46]. Practices will mail the Patient Experience Survey to patients that the early and delayed intervention practices defined as eligible to receive the MOHR assessment two weeks after the patient’s office visit. To enhance survey response rates, surveys will be mailed using a modified Dillman technique in the practice’s envelope with a cover letter from the patient’s provider [47,48]. Study information will be included with the survey, and completion of the survey will be deemed as consent for participating in the survey – our Institutional Review Board approved a waiver of written consent.

### Outcomes assessment

**Figure 4:**
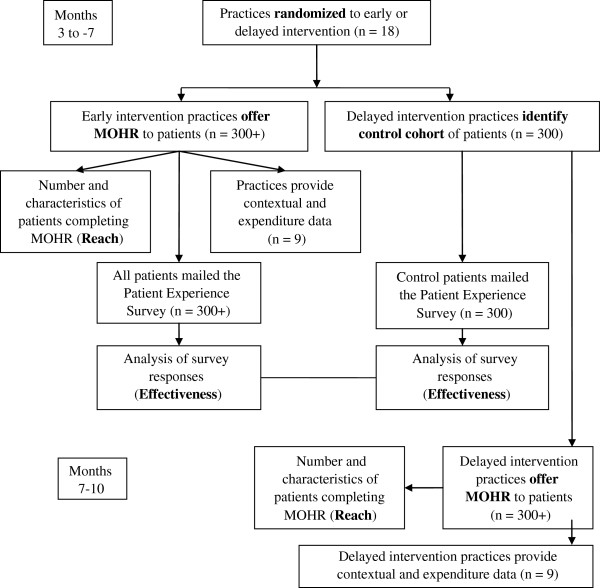
MOHR study CONSORT flow diagram.

The primary outcomes for this study are implementation measures [15]. The measures include the practices’ implementation Reach and Effectiveness [49]. Reach measures include the percent and characteristics of patients that: are defined as eligible to be invited to complete the MOHR assessment; are invited to complete the MOHR assessment; and complete the MOHR assessment. The primary Effectiveness measures are a comparison of early versus delayed intervention practice patient report on whether they: were asked about the health behavior and psychosocial domains; set a goal or action plan with their clinician; and arranged any follow-up contact regarding their plan. As early intervention practices will have implemented the MOHR assessment and delayed intervention practices will not yet have implemented the MOHR assessment, they will respectively serve as intervention and control conditions. The impact on health behaviors and psychosocial problems will be assessed by repeated administration of the MOHR assessment for the subset of patients providing an email address. Patient responses will be compared at baseline and four months after baseline. Multivariate regressions will assess for influences of practice implementation characteristics (*e.g.*, electronic versus paper) and patient demographics on each outcome. The overall CONSORT diagram depicting study participation and data capture are depicted in Figure 4.

### Power and sample size analysis

To determine power, we focused on the patient report as to whether any practice personnel worked with her/him to set specific goals for change for the 10 behavior and mental health topics. For the base power analysis, we assumed 9 matched pairs of sites, 300 patients mailed the Patient Experience Survey from each site, and a 50% response rate (2,700 total respondents). Imputation analyses will be conducted to evaluate the impact of missing data, but here we assume the loss of up to 50% of the sample for power calculation purposes. For the first power analysis, we focus on the binary event that at least one goal was established (as opposed to none). Assuming a delayed intervention response rate of 0.3, there is 90% power to detect a 17.5% improvement in the early versus delayed intervention group, and 80% power to detect a 15% improvement.

For the second power analyses, we focus on the continuous outcome of the number of goals established using a hierarchical analysis of covariance. We adjust for any baseline differences in variables related to the outcome as well as clustering effects of patients within clinic. Assuming an intraclass correlation coefficient (ICC) of 0.03, there is 90% power to detect a between conditions effect size of 0.47 with 9 sites per treatment and a 50% patient response rate, and 80% power to detect an effect size of 0.40. Assuming a larger ICC of 0.05, we have 80% power to detect an effect size of 0.51 and 90% power to detect a difference of 0.60, given the assumptions above. All power analyses were performed using the ‘Optimal Design Software’ package (version 3.0).

### Contextual assessments

An important aspect of a pragmatic trial is that it is implemented in ‘real-world’ contexts, making it essential that these contexts be described to understand differential outcomes [50]. Accordingly, local research teams will collect data on multiple levels of contextual factors [51-53]. Levels will include national/state/local, community, healthcare system, practice and patient [54]. Contextual factors will include practice culture and staffing, patient panel characteristics, community characteristics and resources, and organizational features [55]. Local research teams will collect relevant data through structured interviews with clinicians and through field observations of clinical, social and organizational processes and interactions as practices field the MOHR assessment at the baseline, middle and end of the intervention to assess changes in context. Quantifiable characteristics will be incorporated from initial practice descriptions made during recruitment. Teams will use a standardized template to systematically elicit and record contextual data.

### Cost assessment

The MOHR cost assessment will evaluate both the implementation costs and the replication costs using cost assessment procedures that are minimally intrusive and burdensome [19]. Using standardized a procedure, local research teams will collect each practice’s cost estimate following brief training. This person will use a combination of procedures, including observation of the intervention and brief standardized structured questions of key practice implementation staff. Cost data will be collected at two time points – early in the implementation and once a steady state has been reached. Calculations will be made using established expenditure methods, and replication costs will be categorized based upon different practice and implementation characteristics [56-59]. The cost assessments will be conducted from the practice perspective and will include costs of recruitment, promotion, training, supervision, implementation and follow-up. Data collected during the trial will be used to estimate the cost of a practice replicating the MOHR assessment setting under different conditions and assumptions. Research costs, downstream costs of increased or reduced healthcare utilization, and costs from the patient perspective will not be considered.

### Balancing fidelity and adaption

To maintain internal validity, certain components of the intervention were standardized. All participating practices will implement these components, and fidelity to implementation will be assessed. However, to reflect the pragmatic nature of the trial, practices were encouraged to adapt aspects of the intervention to their workflow and setting [22]. This is necessary because the MOHR study purposively includes practices that vary widely with respect to patients (mostly minority versus non-minority), location (rural versus urban), and EHR and patient portal use. Therefore, to ensure successful implementations as well as high assessment and survey response rates, each practice pair considered its patients’ needs. The elements that needed to be standardized were decided by the overall project steering committee and working groups, while decisions about local tailoring were made by the practice pairs with support and direction from the local research team.

Standard study elements include the MOHR health behavior and psychosocial assessment questions, provision of patient and provider feedback summaries based on the MOHR assessment, goal-setting materials and brief training to support clinicians with counseling, and Patient Experience Survey questions. Practice pairs are collaboratively deciding which patients to target for the MOHR assessment, while attempting to include the largest percentage of adult patients that is feasible; timing of the MOHR assessment (one to two weeks prior to a visit at the patient’s home or immediately prior to a visit at the practice); mode of the MOHR assessment (website or paper), delivery of clinician summary (integrated into EHR, faxed, printed); and mode of administering the Patient Experience Survey (postal, web-based, telephone or a combination).

### Trial status

The MOHR trial began January 2013 and is expected to complete data collection in November 2013. The trial is proceeding according to the timeline outlined in Figure 1. Currently, the early implementation clinics are in the early stages of fielding the MOHR assessment to their patients. The delayed intervention clinics will begin implementation between July and September 2013.

## Discussion

This manuscript describes the need and plans for an important ongoing pragmatic study of the administration and use of patient-reported items in primary care, illustrating key features of implementation design with both standardized and flexible aspects reflecting the dual realities of research and practice. A central tenant throughout the process has been ensuring the relevance of the intervention, measures, and methodologies to stakeholders [10,12].

**Figure 5:**
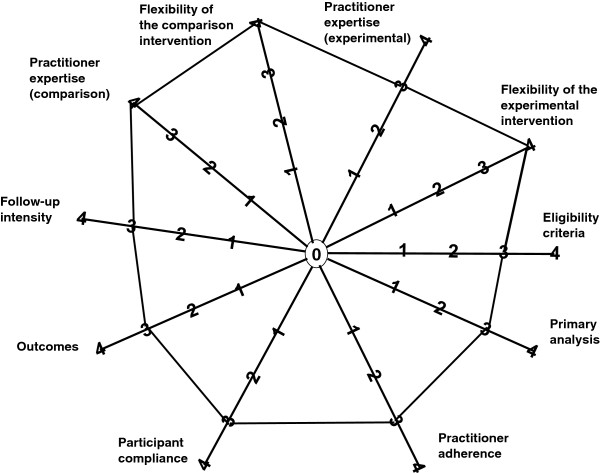
**Depiction of the MOHR study PRECIS characteristics.** The MOHR study design receives completely pragmatic scores for intervention flexibility, control flexibility, and practitioner expertise. The MOHR study design receives the next most pragmatic rating on the remaining seven dimensions.

Figure 5 summarizes the characteristics of the MOHR study on the 10 CONSORT PRECIS dimensions. Overall, the MOHR study is highly pragmatic, receiving completely pragmatic scores on three dimensions (intervention flexibility, control flexibility, and practitioner expertise) and the next most pragmatic rating on the seven remaining dimensions. These scores are substantially more pragmatic than other published trials [60].

Implementation science involves controlled research, in this case a cluster randomized delayed intervention trial, but that is pragmatic and thus more directly relevant to real-world settings [9,61,62]. By engaging all of the stakeholders on the front-end about the importance of healthcare process and outcome metrics for documenting healthcare quality, the results should be more directly usable by both primary care and potential funding and reimbursement agencies. This is especially so when including practical measures that can be embedded into clinical systems [63], applying a feasible and flexible intervention that provides direct feedback based on these practical measures, and assessing outcomes of importance to patient, provider, and policy maker stakeholders, including costs.

This type of science is needed because ‘evidence alone is not enough’ [64,65]. In addition to the best evidence, as in this case recommendations for primary care-based health behavior and psychosocial counseling, other elements are needed [66,67]. As noted in the Evidence Integration Triangle framework, other necessary components for successful implementation are practical measures of progress that can be tracked longitudinally (in MOHR, these are the 17 items), and partnership engagement processes, as illustrated above [64].

A major advantage of the MOHR trial for advancing both scientific and practice frontiers is its diversity of settings. Participating primary care settings include practices that already have adopted state-of-the art e-health technologies for tracking patients’ care, enabling an examination of the merger of patient-centered assessment and intervention approaches and EHRs in high resourced settings [68,69]. On the other end of the continuum, inclusion of a number of community health centers, typically with less access to state-of-the art technologies, has generated problem-solving about the best strategies for conducting assessment and interventions in contexts where staff are challenged to see large caseloads of more diverse, low income/low educated populations with characteristically complex medical and psychosocial needs, and lower levels of health literacy and numeracy [70]. The mantra remains consistent: involving relevant stakeholders is important for both the initial adoption as well as the long-term sustainability of evidence-based patient assessment and intervention. This requires an understanding of the realities of the practice flow, an appreciation for the context of patient lives, and how to design practical assessments and feedback mechanisms that can be implemented with minimal resources.

From a traditional perspective, there are several limitations or weaknesses to the MOHR design. Scientifically, there are several questions the trial will be unable to address. The MOHR assessment will not be linked at the individual level to the follow-up Patient Experiences Survey, and the design selected precludes the examination of the long-term behavioral or biological health impact of our intervention. In contrast to more traditional research studies, the project will not employ expert, highly trained staff in leading academic medical centers to deliver the intervention; rather the intervention will be implemented within real-world settings with tailoring of strategies to meet the needs of each site. Similarly, a decision was made to be as inclusive as possible in the patient intake rather than using strict eligibility criteria selecting for non-complicated, highly motivated patients who often fare better in health promotion programs. Finally, practice participation is not limited to practices with excellent state of the art EHRs serving technology savvy patients; thus, a difficult balance has been sought between maximizing the use of technology whenever possible and developing protocols applicable to a range of settings. All of these decisions could reduce the initial effect size, or produce different results across different contexts, subgroups or settings. However, despite this possibility, we suggest that the project remains a worthwhile investment that will provide invaluable information about ‘what works under what conditions for what groups for what outcomes’— in a relatively short time frame [71].

The study design has many strengths, including the highly pragmatic nature of the protocol, as evidenced by the PRECIS ratings. Given the stakeholder involvement throughout, the results should be highly relevant and sensitive to both the primary care context as well as to the growing emphasis on patient-centered care for improving population health and well being. Anticipating the relevance of the results to diverse primary care practitioners as well as different healthcare settings and networks, the rapid and iterative processes using mixed methods should provide in-depth feedback to more quickly inform practice and policy. Inclusion of data on reach, implementation under different conditions, as well as implementation and replication cost estimates should provide the types of transparent information needed by potential adopting settings. The tools and materials used in the MOHR project, including the standardized brief assessment items, the MOHR automated assessment and feedback tool, and the assessment procedures are all in the public domain and available for others to use or adapt (http://www.MyOwnHealthReport.org). Replications and extensions are encouraged, and especially studies having the scope and funding to evaluate issues such as longer-term sustainability, health behavior changes, and health outcomes.

The MOHR study serves as an example of a pragmatic, participatory design that leverages resources and tailors design elements to a range of local settings, while maintaining the essential elements and scientific rigor of a randomized comparison trial. This design is necessary, given that the MOHR study aims to assess whether it is feasible for a broad range of primary care practices to systematically patient reported health behavior and psychosocial assessments. Outcomes will include all necessary implementation elements including feasibility, impact on care, the influence of setting and context, and the expense of implementation. These findings should be of interest to researchers, practitioners, and policy makers attempting to make healthcare more patient-centered and relevant.

## Competing interests

The authors declare that they have no competing interests.

## Authors’ contributions

All authors collaborated in the design of the study and led or participated in described working groups. AH, BG and RG provided initial drafts of the manuscript and integrated feedback from all authors, the remainder of whom are listed alphabetically. All authors read and approved the final manuscript.
